# Virtual reality vs. imagery: comparing approaches in guided meditation

**DOI:** 10.3389/fpsyg.2024.1472780

**Published:** 2024-11-25

**Authors:** Minkyung Jo, Eunha Kim, Jaeyeon Lee

**Affiliations:** ^1^Life Quality Center, Ajou University, Suweon, Republic of Korea; ^2^Department of Psychology, Ajou University, Suweon, Republic of Korea

**Keywords:** virtual reality, imagery, guided meditation, novice meditators, concentration

## Abstract

**Introduction:**

This study compared the influence of virtual reality (VR)-based and traditional (e.g., imagery-based) guided meditation on stress and concentration levels among South Korean adults. In addition, we examined whether concentration levels differed between individuals who are new to meditation and those who are experienced.

**Methods:**

Seventy participants were randomly assigned to either the VR or imagery condition, where they engaged in breathing and waterfall concentration meditation. Pretest and posttest measures of heart rate (HR), galvanic skin response (GSR), negative affect, and concentration during meditation were recorded.

**Results:**

Both VR- and imagery-based guided meditation significantly reduced HR, GSR, and negative affect. However, no significant differences in outcomes were observed between the two groups. Still, participants in the VR condition reported higher concentration levels during meditation, particularly novice meditators. In addition, qualitative feedback indicated that VR-based meditation was more effective in inducing positive emotions, such as calmness and enjoyment.

**Discussions:**

While both VR- and imagery-based guided meditation effectively reduce stress and negative affect, VR-guided meditation shows promise for enhancing concentration, particularly for novice meditators.

## 1 Introduction

Meditation has received substantial attention in the field of psychology because of its potential benefits for mental health and well-being (Hussain and Bhushan, 2010). Numerous studies have demonstrated that meditation can effectively alleviate various psychological and psychosomatic issues, including stress, symptoms of depression and anxiety, addiction, anger, chronic pain, and insomnia (Bohlmeijer et al., 2010; Chen et al., 2012; Goyal et al., 2010; Hofmann et al., 2010; McGee, 2008; Winbush et al., 2007). Meditation has also been found to enhance self-esteem, self-efficacy, self-control, and regulatory capabilities (Hooper et al., 2024). Owing to these benefits, meditation is increasingly being used in therapeutic settings (Sedlmeier et al., 2012).

Meditation is practiced in various forms with basic techniques such as focusing on one’s breathing patterns or becoming aware of physical sensations (Moral, 2017). Guided meditation is the form used most commonly in therapy, owing to its simplicity and suitability for beginners (Packiasabapathy et al., 2019). Guided meditation involves clients meditating with guidance from a trained therapist or teacher through various media, including in-person instruction, written text, sound recordings, and audiovisual media (Moral, 2017). This process helps clients create sensory perceptions of sights, sounds, and a state of calmness and relaxation. Guided meditation has been employed to treat stress, eating disorders, and depressive thoughts (Kristeller et al., 2006).

Despite its effectiveness, guided meditation presents challenges for both clients and therapists. Studies have found that learning to meditate can be difficult, with clients reporting issues such as distractions caused by the physical environment (e.g., noise, people and the struggle to visualize scenes based on instructions) (Anderson et al., 2019; Lomas et al., 2015a; Lomas et al., 2015b). Therapists face ongoing challenges in helping clients maintain their attention during meditation and visualize relaxing scenes (Lee, 2023). These challenges are compounded by the significant time commitment required for achieving therapeutic effects from guided meditation, typically requiring 20 to 27 h of practice over a period of 8 weeks on average (Goyal et al., 2010). This can be particularly difficult for novice meditators who often experience frustration and decreased motivation due to difficulty in achieving a meditative state (Lindsay and Creswell, 2017; Lutz et al., 2008).

Virtual Reality (VR) technology, an emerging technology used to treat psychological and physical symptoms (e.g., phobia, PSTD, social anxiety, and chronic pain) (Mistry et al., 2020; Emmelkamp et al., 2020; Knaust et al., 2020) may effectively address the limitations of guided meditation (Seabrook et al., 2020). VR-based meditation includes specific guidance alongside immersive virtual environments and background music. Previous studies have revealed that VR can mitigate environmental distractions by offering an immersive, engaging, and controlled environment that allows clients to practice their meditation skills effectively (Navarro-Haro et al., 2017). In addition, the immersive nature of VR creates a strong sense of “presence,” which enhances the fundamental aspects of meditation, such as focusing on breathing and bodily sensations (Liu et al., 2023; Miller et al., 2021; Slater, 2014). However, VR-based meditation is associated with some limitations, such as inadequate video quality, heaviness of VR headsets, and potential motion sickness, which can decrease its effectiveness (Seabrook et al., 2020).

Despite these limitations, previous studies have verified the effectiveness of VR-guided meditation. The participants in these studies reported increased feelings of calmness, relaxation, and happiness (Latif et al., 2020). Significant decreases in blood pressure, heart rate, and levels of pain have also been observed (Han and Jo, 2023; Liu et al., 2023). Specifically, several studies comparing VR-based guided meditation with traditional guided meditation have revealed that VR-based meditation is more effective in improving mindfulness and meditation experiences, sleep quality, and emotion regulation, while also reducing anxiety and depression levels (Beshai et al., 2020; Ma et al., 2023).

Although these studies contribute to the literature by demonstrating the potential of incorporating VR into guided meditation training, several gaps remain. First, many studies did not include meditation training for control groups. Consequently, even if the results indicated that VR-based guided meditation had a more significant impact on participants’ well-being, this effect could be attributed to the meditation itself rather than the VR component. Second, studies that included meditation in their control groups often failed to compare VR-based guided meditation with traditional guided meditation under identical conditions, leading to potential confounding factors.

For example, Navarro-Haro et al. (2019) compared an experimental group that utilized VR with a control group that did not and found significant reductions in symptoms of anxiety and depression in both groups. However, one limitation of the study was the allocation of time (15 min) dedicated to VR meditation compared with the total program duration (105 min). This discrepancy makes it challenging to determine whether the observed effects were due to the VR component or other elements of the program. In addition, Yildirim and O’Grady (2020) compared VR-based mindfulness with non-VR audio-guided mindfulness; however, the audio guidance provided to the participants in the two groups was not identical. To robustly demonstrate the effects of VR-based guided meditation, further studies that directly compare VR-based guided meditation with traditional-guided meditation under identical conditions is necessary. Both groups should receive the same amount of meditation time and similar program content, with the only variable being the presence or absence of a VR component.

Additionally, previous studies do not examine third variables that might influence the effectiveness of VR-based guided meditation compared with traditional guided meditation. Investigating these variables would enable therapists to determine the type of guided meditation that is more beneficial for their clients and tailor interventions accordingly. For example, evidence suggests that VR can enhance attention during meditation for novice meditators who often struggle to maintain focus (Anderson et al., 2019; Lutz et al., 2008). However, no study has examined whether VR-based guided meditation is more effective for novices than for experienced meditators.

To address the research gap, this study directly compared VR-based guided meditation with traditional guided meditation (e.g., imagery-based guided meditation) among South Korean adults. In addition, it investigated whether levels of concentration during VR-based guided meditation and imagery-based guided meditation would differ between individuals who are new to meditation and those who are experienced. The South Korean context is particularly relevant due to the country’s high suicide rate and long working hours (OECD, 2024), which have facilitated a growing culture of “well-being” and heightened interest in meditation (Choi, 2022). To measure the relative effectiveness of VR-based- and imagery-based guided meditation, we utilized both self-report measures of negative affect and concentration, along with physiological data, such as heart rate (HR) and galvanic skin response (GSR), as indicators of stress. Previous studies on meditation have widely used HR and GSR as physiological markers of stress (e.g., Das and Anand, 2012; King et al., 2024; Liu et al., 2023). The inclusion of physiological data provides objective metrics to complement subjective self-reports and is suitable for measuring the effectiveness of short meditation sessions.

Based on the effectiveness of VR-based- and imagery-based guided meditation, we predicted the following: (1) H1: Both VR- and imagery-based guided meditation will decrease the levels of HR, GSR, and negative affect from pretest to posttest. Second, because VR provides more presence and immersion in an environment than imagery-based guided meditation, we anticipated the following: (2) H2: VR-based guided meditation will decrease the levels of HR, GSR, and negative affect more compared with imagery-based guided meditation from pretest to posttest; and (3) H3: Levels of concentration during VR-based guided meditation will be higher than during imagery-based guided meditation. Finally, because maintaining concentration during imagery-based guided meditation is difficult for those who are new to meditation, we predicted (4) H4: Concentration levels during VR-based guided meditation will be higher for novice meditators, whereas concentration levels during imagery-based guided meditation will be higher for experienced meditators.

## 2 Materials and methods

### 2.1 Participants

The desired sample size was determined using a G*Power analysis. With parameters set to *f* = 0.25, *α* = 0.05, and Power = 0.9, G*Power recommended a sample size of 64 participants. To account for potential dropouts owing to cybersickness and other reasons, we recruited 70 participants. All participants met the eligibility criteria of being at least 18 years old and not currently receiving psychiatric care. A total of 36 participants were randomly allocated to the VR-based guided meditation condition (VR condition) and 34 participants to the imagery-based guided meditation condition (imagery condition). Given the study objective of comparing novice and experienced meditators, we used a between-subjects design. We were concerned that, in a repeated-measures design, the experience of participating in the first condition (e.g., VR meditation) may influence participants’ responses in the second condition (e.g., non-VR meditation) due to learning effects or increased familiarity with meditation techniques. This could bias the results by introducing unintended carryover effects, thereby compromising the comparisons’ validity. Therefore, to minimize the risk of these confounding effects, a between-subjects design was deemed more appropriate. The baseline characteristics of the participants are summarized in Table 1.

**Table 1:** Baseline characteristics of participants.

Background variables	VR condition		Imagery condition		Total	
	*n*	%	*n*	%	*n*	%
Gender						
Male	14	20.0	16	22.9	30	42.9
Female	22	31.4	18	25.7	40	57.1
Previous meditation experience						
Yes	17	24.3	12	17.1	29	41.4
No	19	27.1	22	31.4	41	58.6
Age, *M* (*SD*); range	30.81 (11.65); 20–62		26.81 (9.55); 20–62		28.93 (10.79); 20–62	

### 2.2 Procedure

After receiving approval from the institutional review board (#202403-HB-003), we recruited participants by posting recruitment flyers on college campuses. Eligibility was determined via email or telephone screening. Upon arrival, the participants were individually escorted to the testing room and informed about the study’s purpose, procedure, the voluntary nature of participation, and confidentiality. The participants assigned to the VR condition were also informed about the potential side effects of VR. After signing the consent form, a pretest was conducted to measure their HR, GSR, negative affect, and previous meditation experience. The experiment session was initiated and lasted for 18 min. During the experiment, efforts were made to minimize distraction in both conditions, such as having the researchers sit out of sight of the participants.

Participants in the VR condition were instructed to sit comfortably and wear the MetaQuest Pro VR all-in-one device with two VR programs: Guided Meditation^1^ and TRIPP.^2^ These platforms provide immersive meditation experiences designed to reduce external distractions and facilitate mental relaxation while exploring breathtaking landscapes and virtual worlds (Casu et al., 2024). The English audio guidance provided by these programs was muted, and the participants received verbal guidance in Korean from the researchers. The participants in the imagery condition were asked to sit comfortably with their eyes closed, adopt a relaxed posture, and visualize scenes in response to the researchers’ verbal guidance. Verbal guidance in both conditions consisted of two parts (breathing meditation and waterfall concentration meditation) and was delivered using prerecorded audio instructions (see Table 2).

**Table 2:** Verbal guidance during meditation.

	VR condition	Imagery condition
Breathing meditation	“Breathe slowly and deeply in accordance with the flow of light seen in front of you. Concentrate on the breathing.”	“Breathe slowly and deeply. Concentrate on the breathing.”
Waterfall concentration meditation	“Focus on a cascading waterfall and direct your attention fully to the waterfall. Let it become the sole object of your concentration. If your mind starts to wander or thoughts arise, gently bring your focus back to the cascading waterfall. Let go of any worries and anxieties, imagining them flowing away like water.”	“Imagine a cascading waterfall in your mind’s eye. Picture it vividly: the cascading water, the sound it makes, and the mist that surrounds it. Direct your attention fully to the cascading waterfall image you have created. Let it become the sole object of your concentration. If your mind starts to wander or thoughts arise gently bring your focus back to the cascading waterfall. Let go of any worries and anxieties, imaging them flowing away like water.”

In the VR condition, participants first engaged with Guided Meditation, followed by TRIPP. This order was predetermined to ease participants into the VR experience, beginning with general guided meditation before transitioning to the more interactive features of TRIPP. Specifically, participants completed breathing meditation using Guided Meditation and then moved on to waterfall concentration meditation in TRIPP. Those in the imagery condition followed the same sequence. Following the experimental session, all participants completed posttest measures of HR, GSR, negative affect, and concentration during meditation.

Breathing and nature-based meditation (here, the waterfall was used as the natural environment) are widely recognized techniques for enhancing mindfulness and reducing stress. Breathing meditation involves focusing on one’s breath to remain grounded in the present moment (Komariah et al., 2022; Paul et al., 2007). Meanwhile, nature-based meditation immerses individuals in natural environments, fostering a deeper connection to nature and promoting inner peace (Owens and Bunce, 2022; Ray et al., 2021).

### 2.3 Measure

#### 2.3.1 Previous experience with meditation

Previous experience with meditation was measured during the pretest phase by asking participants, “Have you learned or had experience with meditation in the past?” (yes/no). Those who responded “Yes” were classified as “novice meditators” while those who responded “No” were classified as “experienced meditators.”

#### 2.3.2 HR

HR was measured using photoplethysmography (PPG) at both pretest and posttest. A PPG sensor (Model: Ubpulse T1, Pulse Analyzer, KFDA Citification No. 11–1,296, LAXTHA Ltd., Korea) was applied to the left index finger. HR serves as an indicator of physiological stress responses in human participants, with increases in HR associated with higher stress levels (Mostajeran et al., 2021).

#### 2.3.3 GSR (Galvanic Skin Response)

GSR was measured using a GSR sensor manufactured by NeuLog (Israel) at both pretest and posttest. The sensor, equipped with ring-type electrodes, was placed between the left index and middle fingers, and connected to a computer via a USB port for data transmission. GSR measures changes in skin electrical conductivity, which reflects the production of ionic sweat by the skin glands in response to stimuli. This physiological response is considered an indicator of localized phasic arousal processes and has been widely utilized as a measure of stress (Mostajeran et al., 2021; Vossel and Zimmer, 1992). Higher skin conductance indicates a higher stress level.

#### 2.3.4 Negative affect scale

Negative affect was measured using the negative affect subscale of the Positive and Negative Affect Schedule (PANAS; Watson et al., 1988) at both pretest and posttest. This subscale consists of 10 items that measure negative emotions. Each item is rated on a 6-point Likert-type scale ranging from 0 (not at all) to 5 (extremely), with high scores indicating higher levels of negative affect. Participants rated their negative affect “right at the moment.” The South Korean version of the PANAS has demonstrated good reliability and internal consistency (Cronbach’s alpha 0.83; Park and Lee, 2016). In this study, Cronbach’s alpha for the negative affect subscale was 0.83.

#### 2.3.5 Concentration during meditation

The concentration level during meditation was measured posttest using a three-item scale that we developed based on the literature. The items were (1) “I was able to focus well on my breath during meditation,” (2)” I did not get distracted by other thoughts during meditation,” and (3) Even if I found myself having other thoughts during meditation, I found it easy to quickly return to meditation.” Each item is rated on an 8-point Likert-type scale ranging from 0 (not at all) to 7 (extremely), with high scores indicating higher levels of concentration during meditation. Cronbach’s alpha for these items was 0.87.

#### 2.3.6 Subjective opinions on the meditation experience

Participants were asked to share their experiences of VR or imagery-based meditation in response to an open-ended question, “Please freely describe your experience of meditation in this study.”

## 3 Results

### 3.1 Baseline characteristics

Baseline characteristics between the VR and imagery conditions, including age and outcome measures (i.e., HR, GSR, negative affect), were compared using *t-*tests. No significant differences were observed between the conditions (Table 3). In addition, chi-square tests revealed no significant differences in gender distribution or previous meditation experience between the conditions.

**Table 3:** Comparison of the VR and imagery conditions at baseline.

	VR condition (*n* = 36)		Imagery condition (*n* = 34)			
	*M*	*SD*	*M*	*SD*	*t*	*p*
Age	30.81	11.65	26.81	9.55	1.510	0.136
HR	83.89	2.033	82.18	13.44	0.562	0.576
GSR	16830.14	8203.59	16971.0	8797.26	−0.069	0.945
Negative affect	9.11	6.44	9.00	5.56	0.079	0.937

### 3.2 Hypotheses 1 and 2: analysis of HR, GSR, and negative affect

To test the first and second hypotheses, we conducted repeated-measures multivariate analyses of variance (MANOVAs) on HR, GSR, and negative affect. The means, standard deviations, and MANOVA statistics based on condition (i.e., VR, imagery) and time (i.e., pretest, posttest) are presented in Table 4. While the main effects of the conditions on HR, GSR, and negative affect were not significant, the main effects of time were significant. No significant interactions were observed between the condition and time. Furthermore, paired-sample *t*-tests were conducted to examine pretest/posttest differences (see Table 5) and differences between the VR and imagery conditions at the posttest (see Table 6). The results indicated significant decreases in HR, GSR, and negative affect from pretest to posttest for both the VR and imagery conditions. The effect sizes at posttest were also calculated using Cohen’s *d*. An effect size greater than 1.2 is considered very large, while 0.56 ~ 1.2 is considered large, 0.33 ~ 0.55 moderate, and less than 0.33 small (Lipsey and Wilson, 1993). The results indicated moderate to large effects (HR: VR d = 0.46, imagery d = 0.27; GSR: VR d = 0.60, imagery d = 0.71; negative affect: VR d = 1.29, imagery d = 1.46).

**Table 4:** MANOVA statistics for the outcome variables as a function of condition by time.

			HR	GSR	Negative affect
VR condition	Pre	*M (SD)*	83.89 (12.03)	16830.14 (8203.59)	9.11 (6.44)
	Post	*M (SD)*	78.81 (9.61)	12122.67 (7243.38)	2.48 (3.26)
Imagery condition	Pre	*M (SD)*	82.18 (13.44)	16971.00 (8797.26)	9.00 (5.56)
	Post	*M (SD)*	78.85 (10.23)	11122.21 (7442.21)	2.47 (2.97)
MANOVA statistics	Condition^a^	*F* (1, 68)	0.09	0.06	0.004
	*p*		0.754	0.805	0.948
	Time^a^	F (1, 68)	37.64^***^	47.20^***^	122.23^***^
	*p*		<0.001	<0.001	<0.001
	Condition x Time^b^	F (1, 68)	1.65	0.552	0.007
	*p*		0.203	0.460	0.934

MANOVA, multivariate analysis of variance.***p < 0.001.

a Main effect.

b Interaction effect.

**Table 5:** Paired-Sample *t*-tests for pre-post comparison.

		HR	GSR	Negative affect
VR condition	*t*	5.390^***^	5.646^***^	7.457^***^
	*p*	<0.001	<0.001	<0.001
Imagery condition	*t*	3.339^**^	4.458^***^	8.287^***^
	*p*	0.002	<0.001	<0.001

^**^p < 0.01; ^***^p < 0.001.

**Table 6:** Independent sample *t*-tests for VR-imagery comparison at posttest.

	HR	GSR	Negative affect
*t*	−0.020	0.570	0.040
*p*	0.945	0.793	0.585

### 3.3 Hypothesis 3: analysis of concentration during meditation

An independent samples *t*-test was conducted to compare the levels of concentration during meditation between the VR and imagery conditions. The results reveal a significant difference (Table 7), with the VR condition demonstrating higher concentrations than the imagery condition.

**Table 7:** Level of concentration during meditation according to the type of meditation.

	*M*	*SD*	*t*	*p*
VR condition	15.91	2.90	2.80^**^	0.007
Imagery condition	13.64	3.83		

**p < 0.01.

### 3.4 Hypothesis 4: novice meditators vs. experienced meditators

We performed a one-way ANOVA to examine the differences in HR, GSR, negative affect, and concentration during meditation in terms of condition and previous meditation experience. Significant differences were found among the four groups in concentration scores (see Table 8). The post-hoc Scheffé test indicated that participants with no previous meditation experience in the VR condition (Group 2) reported significantly higher levels of concentration during meditation than those in the imagery condition (Group 4).

**Table 8:** One-way analysis on outcome variables by condition and previous meditation experience.

	VR condition		Imagery condition		*F*	Scheffé
	Meditation experience	No meditation experience	Meditation experience	No meditation experience		
	Group 1^a^	Group 2 ^b^	Group 3 ^c^	Group 4 ^d^		
	*M(SD)*	*M(SD)*	*M(SD)*	*M(SD)*		
HS	79.29 (9.05)	78.37 (10.32)	77.75 (8.95)	79.45 (11.02)	0.10	
GSR	11,592.00 (5,490.50)	12,597.47 (8,643.98)	10,632.67 (7,013.06)	11,389.23 (7,814.54)	0.18	
Negative affect	3.35(3.88)	1.73(2.32)	1.91(3.08)	2.77(2.94)	1.02	
Concentration	15.11(3.19)	16.63(2.47)	14.08(3.94)	13.40(3.85)	3.32^*^	(b) > (d)

^*^p < 0.05.a=Group 1, b=Group 2, c=Group 3, d=Group 4.

### 3.5 Qualitative analyses

The participants’ qualitative responses regarding their meditation experiences in both conditions were analyzed using interpretive phenomenological analysis (IPA). IPA was chosen because of its focus on the lived experience of individuals and its emphasis on empathic interpretation of participant narratives (Smith et al., 2009). The results for the VR and imagery conditions are summarized in Tables 9, 10, respectively. Participants in both conditions reported positive outcomes of meditation, such as relaxation and enhanced focus on breathing. Those in the VR condition highlighted the benefits of an immersive environment with appropriate visual and auditory stimuli. However, they also noted technical issues including poor image quality and discomfort caused by wearing the VR headset.

**Table 9:** Categories and subcategories in the VR condition.

Category	Subcategory	Illustrative quotes
Facilitated immersion in meditation	Experiencing a true meditative state	“When I used to meditate in classes before, I just felt sleepy. However, while using VR for breathing meditation, I realized, ‘Ah, this is what it feels like to be truly immersed in meditation.’”
	Helpful visual and auditory stimuli	“It was interesting that even though my actual surroundings had not changed, I could immerse myself in the experience through relaxing sounds and natural environment scenes.”
	Disappearance of distractions	“Just following along with the guidance on screen comfortably, it felt like I had some time without wandering thoughts for the first time in a while.”
Positive emotions	Calm and comfortable	“It was a comfortable, restful experience.” “I felt lighter and relieved, and my mood improved.”
	Enjoyed	“It was enjoyable. I appreciated being able to meditate in various places and situations using VR.”
Difficulty focusing on meditation	The lack of presence	“The immersion was hindered by the VR’s image quality and the visible mechanical parts around me.”
	Physical discomfort	“The screen’s shaking and the pressure from the VR device on my head were distracting during meditation.”

**Table 10:** Categories and subcategories in the imagery condition.

Category	Subcategory	Illustrative quotes
Positive effect of meditation	Tension relief and a sense of relaxation	“I felt a sense of relaxation setting in. I was so relaxed that I almost dozed off.”
	Mind being cleared up	“My cluttered mind cleared up, and I feel my tired body and mind recovering.”
Difficulty focusing on meditation	Too many distractions	“I found it challenging to focus on my breathing or visualize a waterfall. Despite my efforts to ignore distractions, they persisted. I tried to focus on meditation, but distracting thoughts kept coming to my mind.”
	No visual and auditory aids for meditation	“It would have been helpful to have sound assistance to enhance visualization of scenes such as waterfalls or forests.”

## 4 Discussion

Our study aimed to investigate the effectiveness of VR-based-and imagery-based guided meditation among South Korean adults, focusing on physiological and subjective outcomes (HR, GSR, negative affect, and concentration level). We discuss below the major findings in relation to our hypotheses and the existing literature.

### 4.1 Major findings

The results indicated that both VR and imagery meditation effectively decreased HR, GSR, and negative affect from baseline scores. Thus, our first hypothesis, which predicted a significant reduction in these measures under both conditions, was confirmed. Although the effectiveness of VR-based meditation has not been extensively documented, previous studies have reported encouraging results (Flores et al., 2018; Gomez et al., 2017). Our findings align with these studies, suggesting the benefits of VR-based guided meditation (Failla et al., 2022; Navarro-Haro et al., 2017) for stress reduction and mood improvement among South Korean adults in a controlled experiment.

However, our second hypothesis, which predicted that VR-based guided meditation would outperform imagery-based guided meditation in reducing HR, GSR, and negative affect, was not supported. Our findings contrast with those of prior research, indicating the potential superiority of VR in reducing stress and negative affect (Navarro-Haro et al., 2019; Ma et al., 2023). Nevertheless, some previous findings have also suggested that the effectiveness of VR-based-and non-VR-based meditation is not significantly different (King et al., 2024). One possible explanation for this inconsistency is the initial arousal induced by the VR experience, which can diminish the immediate stress-reducing effects. For example, the participants in this study who are less exposed to VR may experience heightened anticipatory anxiety when using VR equipment. In support of this explanation, prior research has suggested that VR is inherently arousing (Lombard and Ditton, 1997), which may create a slight increase in arousal at the beginning of the VR meditation.

Despite the similar effects of VR and imagery meditation on HR, GSR, and negative affect, qualitative feedback from participants suggested that VR-based guided meditation (not imagery-based guided meditation) was effective in inducing positive emotions. Although we did not measure changes in positive emotion following either meditation method, participants from the VR condition qualitatively described feeling “calm and comfortable” and reported that they “enjoyed” the experience. While the primary goal of meditation is not to foster a positive mood, the ability of VR-based meditation to facilitate positive emotions could be beneficial in mitigating experiential challenges related to negative emotions (Garland et al., 2015). Several studies show that VR-based meditation interventions can effectively induce positive affect, such as wonder, joy, peace, inspiration, and interest (Miller et al., 2021; Seabrook et al., 2020). However, it is critical to consider that positive affect might result from the use of VR itself; thus, the content presented in the VR environment to participants is likely to play a crucial role (Seabrook et al., 2020). In this study, positive affect may have stemmed from the simulated waterfall environment, which participants found calming, comfortable, and enjoyable.

Third, our third hypothesis was supported, indicating that participants in the VR condition demonstrated higher concentration during meditation than those in the imagery condition. The participants reported that the increased concentration observed in the VR condition was due to the immersive nature of the VR environment. Specifically, participants noted that these environments blocked the user’s view of the real world and helped them focus their attention on meditation, which is congruent with observations from previous studies (Navarro-Haro et al., 2019). In addition, the availability of visual and auditory stimuli within a VR environment helps minimize mind wandering (Costa et al., 2019; Navarro-Haro et al., 2017). Therefore, for individuals who have difficulty maintaining focus during meditation, VR-based meditation offers a promising alternative tool for reducing stress levels and negative affect.

Furthermore, our fourth hypothesis was supported. Novice meditators in the VR condition reported significantly higher concentration levels than those in the imagery condition. Thus, VR-guided meditation may be especially beneficial for individuals who are new to meditation, as the immersive nature of VR helps maintain focus. Studies show that maintaining continuous attention on a specific object is a challenging skill for novices (Anderson et al., 2019; Lutz et al., 2008). In contrast, experienced meditators may already possess the concentration skills necessary to engage in various meditation methods. These findings align with prior research on VR’s potential to enhance attention. However, they also contradict research that emphasizes the benefits of mindfulness training primarily for experienced meditators in stressful immersive virtual environments (Crescentini et al., 2016). Future research can explore how VR-guided meditation may be tailored specifically to novice meditators to maximize its potential for supporting the development of concentration and mindfulness skills. Scholars can also investigate its long-term effects compared to traditional mindfulness training programs.

An important aspect of the current study was the use of single-session VR interventions. While these interventions are shorter in duration, they offer several unique benefits. Single-session interventions can provide immediate stress relief and relaxation. Consequently, they can be particularly useful for individuals seeking quick and accessible ways to manage acute stress or emotional distress (Rubin et al., 2024). These interventions are also convenient for those who may have limited time or may not be ready to commit to longer programs, such as mindfulness-based stress reduction (MBSR). MBSR is an evidence-based, eight-week program designed to teach mindfulness mediation (Kabat-Zinn, 1990). Although initially developed for stress management, MBSR has since been shown to effectively address anxiety, depression, pain, and somatic symptoms (Niazi and Niazi, 2011).

While single-session interventions are effective for short-term stress relief and may serve as an entry point for further mindfulness practices, they do not offer the sustained benefits associated with multi-session programs such as MBSR. Future research should investigate how VR-based mindfulness interventions can be structured into multi-session formats like MBSR and whether these adaptations can produce the same depth of long-term benefits.

### 4.2 Implications for clinical practice

The findings of this study hold several implications for clinical practice. First, we found that VR-based- and imagery-based guided meditation were equally effective in reducing physiological stress markers and negative affect. This equivalence is noteworthy for individuals who find imagery-based meditation difficult and seek alternatives to traditional practices. Clinicians are advised to explore their clients’ comfort levels in using meditation techniques, their proficiency in skills such as imagination or visualization, and their previous experience with VR before deciding on a method. Understanding these factors can help to tailor meditation interventions that suit individual preferences and needs. Specifically, clinicians are encouraged to consider that individual differences in mental imagery ability can influence participants’ engagement with traditional imagery-based mindfulness practices, as not everyone possesses strong visualization skills. Individuals with lower mental imagery capabilities may find it difficult to fully immerse themselves in guided meditations that rely heavily on visualizing calming scenes (Cui et al., 2007). This may explain some variations in participants’ responses to such interventions. Overall, these findings underscore the potential benefits of incorporating VR into mindfulness and relaxation interventions.

Second, our study highlighted the significant advantage of VR in enhancing concentration during meditation. This finding suggests potential benefits for clients experiencing attention problems, such as anxiety symptoms or ADHD. For these clients, efforts to generate and sustain specific images during imagery-based meditation can paradoxically increase rather than reduce stress. Third, our findings underscore the importance of adapting meditation interventions based on the client’s previous experience with meditation. The novice meditators in our study reported significantly higher levels of concentration during VR-based meditation than their counterparts who used imagery-based methods. This suggests that VR can be particularly beneficial for individuals who are new to meditation and may find traditional techniques challenging to initiate and maintain. Clinicians should consider using VR-based meditation with novice meditators to facilitate initial engagement and concentration.

### 4.3 Limitations and directions for future research

First, we used a self-report, three-item scale that we developed to measure concentration levels during meditation at the posttest. Future research could use established scales such as the Mindful Attention Awareness Scale (MAAS) (Brown and Ryan, 2003) or the Five Facet Mindfulness Questionnaire (FFMQ) (Baer et al., 2006) to measure concentration and mindfulness during meditation. These scales provide a more comprehensive assessment of participants’ mindfulness experiences than our three-item scale. Scholars may also benefit from incorporating more objective measures, such as neuroimaging techniques (e.g., fMRI and EEG), to measure concentration during meditation.

Second, although this study examined the short-term effects of meditation, longitudinal research is necessary to evaluate the temporal sustainability of these benefits. Long-term follow-up and larger randomized controlled trials are essential to determine whether VR-based guided meditation leads to long-term improvements in mental health outcomes compared with traditional methods.

Third, we did not compare the various types of VR programs. Certain VR environments may be more effective than others in lowering HR, GSR, and negative affect. Future studies should investigate how different VR elements may influence these outcome measures (King et al., 2024).

Fourth, heart rate variability (HRV) data were not included in this study. HRV is a valuable physiological marker for assessing autonomic nervous system regulation and stress responses. Hence, it is an important measure for evaluating the effectiveness of psychological interventions such as meditation. Future studies should include HRV measurements to provide a more comprehensive understanding of both the psychological and physiological effects of meditation practices.

Fifth, we primarily focused on measuring negative affect (NA) and did not include positive affect (PA) as an outcome variable. While reducing NA is important, research has demonstrated that VR-based meditation may be particularly effective in enhancing PA, including emotions such as joy, peace, and inspiration (Miller et al., 2021; Seabrook et al., 2020). Future studies should consider incorporating both NA and PA to more comprehensively capture the emotional effects of VR meditation interventions. In addition, we utilized only visual and auditory cues. Integrating sensory stimulation, such as olfactory stimuli, into VR meditation interventions may further enhance the meditative experience (Pizzoli et al., 2022). For instance, incorporating pleasant or familiar scents into VR environments could foster deeper emotional engagement and relaxation during mindfulness practices. Future research should explore the potential of olfactory stimuli in creating multisensory VR meditation experiences.

Finally, qualitative feedback from the VR participants indicated that the discomfort caused by the VR headset was a source of distraction. This suggests that participant comfort should be prioritized when selecting a VR headset to enhance mindfulness exercises. Future studies could benefit from allowing participants additional time to habituate themselves to and adjust their VR headsets.

## 5 Conclusion

In conclusion, our study is one of the first to provide empirical evidence that VR-based guided meditation is as effective as imagery-based guided meditation among South Korean adults. Previous investigations of VR-based guided meditation have not used a controlled experimental design, especially regarding its effectiveness when compared with traditional meditation. This is one of the strengths of this study. In addition, this study shows that VR-based guided meditation demonstrated superior effectiveness in enhancing concentration during meditation, which is particularly beneficial for novice meditators. These findings highlight the potential of VR technology in innovating therapeutic interventions that can promote mental well-being through immersive and engaging experiences.

## Data Availability

The datasets presented in this article are not readily available because the research data is restricted to access by authorized personnel only. The data cannot be shared or used for purposes beyond the scope of the study. Requests to access the datasets should be directed to Eunha Kim, eunkim@ajou.ac.kr.

## Footnotes

^1^ http://guidedvr.com/

^2^ https://www.tripp.com/
